# Surface Thermo-Dynamic Characterization of Poly (Vinylidene Chloride-Co-Acrylonitrile) (P(VDC-co-AN)) Using Inverse-Gas Chromatography and Investigation of Visual Traits Using Computer Vision Image Processing Algorithms

**DOI:** 10.3390/polym12081631

**Published:** 2020-07-23

**Authors:** Vijay Kakani, Hakil Kim, Praveen Kumar Basivi, Visweswara Rao Pasupuleti

**Affiliations:** 1 Information and Communication Engineering, Inha University, 100 inharo, Nam-gu Incheon 22212, Korea; vjkakani@inha.ac.kr (V.K.); hikim@inha.ac.kr (H.K.); 2 Department of Chemistry, Sri Venkateswara University, Tirupati, Andhra Pradesh 517502, India; 3 Department of Biomedical Sciences & Therapeutics, Faculty of Medicine and Health Sciences, University Malaysia Sabah, Kota Kinabalu Sabah 88400, Malaysia; 4 Department of Biochemistry, Faculty of Medicine and Health Sciences, Abdurrab University, Jl Riau Ujung No. 73, Pekanbaru 28292, Riau, Indonesia

**Keywords:** Poly(vinylidene chloride-co-acrylonitrile) (P(VDC-co-AN )), thermo-dynamic surface characterization, surface free energy, inverse gas chromatography, visual traits, computer vision and image processing

## Abstract

The Inverse Gas Chromatography (IGC) technique has been employed for the surface thermo-dynamic characterization of the polymer Poly(vinylidene chloride-co-acrylonitrile) (P(VDC-co-AN)) in its pure form. IGC attributes, such as London dispersive surface energy, Gibbs free energy, and Guttman Lewis acid-base parameters were analyzed for the polymer (P(VDC-co-AN)). The London dispersive surface free energy ($\gamma_{S}^{L}$) was calculated using the Schultz and Dorris–Gray method. The maximum surface energy value of (P(VDC-co-AN )) is found to be 29.93 mJ·m${}^{-2}$ and 24.15 mJ·m${}^{-2}$ in both methods respectively. In our analysis, it is observed that the $\gamma_{S}^{L}$ values decline linearly with an increase in temperature. The Guttman–Lewis acid-base parameter $K_{a},K_{b}$ values were estimated to be 0.13 and 0.49. Additionally, the surface character S value and the correlation coefficient were estimated to be 3.77 and 0.98 respectively. After the thermo-dynamic surface characterization, the (P(VDC-co-AN)) polymer overall surface character is found to be basic. The substantial results revealed that the (P(VDC-co-AN)) polymer surface contains more basic sites than acidic sites and, hence, can closely associate in acidic media. Additionally, visual traits of the polymer (P(VDC-co-AN)) were investigated by employing Computer Vision and Image Processing (CVIP) techniques on Scanning Electron Microscopy (SEM) images captured at resolutions ×50, ×200 and ×500. Several visual traits, such as intricate patterns, surface morphology, texture/roughness, particle area distribution ($D_{A}$), directionality ($D_{P}$), mean average particle area ($\mu_{avg}$) and mean average particle standard deviation ($\sigma_{avg}$), were investigated on the polymer’s purest form. This collective study facilitates the researches to explore the pure form of the polymer Poly(vinylidene chloride-co-acrylonitrile) (P(VDC-co-AN )) in both chemical and visual perspective.

## 1. Introduction

### 1.1. Purpose of Study

The primary goal of this study is to estimate the surface thermodynamic properties of the polymer (P(VDC-co-AN)) in its pure form using inverse gas chromatography techniques. In addition to that, the secondary intention is to explore the visual traits of polymer (P(VDC-co-AN)) using SEM images and CVIP techniques. Eventually, this study anticipates that the investigated aspects of this work might be obliging for future studies working on the polymer (P(VDC-co-AN)). Generally, polymers, such as Polyvinyl Chloride (PVC) and other variants of PVC, have been employed in industrial applications and other fields of research for decades [1,2,3,4]. Especially, when treated properly and shaped, the PVC and its variants can attain feasible properties that can be utilized in a variety of applications that are related to blood bags, healthcare, automobiles, electronics, and many more rigid and flexible products [5,6,7,8]. The variants of PVC can be used to blend with various other materials for better durability and effectiveness still being an affordable product for many applications [9,10]. One among such variants is Poly (vinylidene chloride-co-acrylonitrile (P(VDC-co-AN)), which has been considered for Inverse Gas Chromatography (IGC) based surface thermodynamic characterization in this study. (P(VDC-co-AN)) has been employed in many engineering applications related to optoelectronic devices, such as Organic Light-Emitting Diodes, Organic Thin-Film Transistors due to its outstanding barrier properties and transparency [11,12]. Applications, such as optoelectronic devices, require higher barrier properties, because the inner mechanics must avert the incursion of water and oxygen molecules into the light-emitting materials [13]. This vacuum system of using organic and inorganic materials is expensive and not robust against mechanical deformation and thermal shock. The use of (P(VDC-co-AN)) in the manufacturing of optoelectronic devices is more desirable due to its robust physical properties, which increases the lifespan and quality of the optoelectronic devices [14]. Furthermore, the polymer (P(VDC-co-AN)) incorporated with silica increases the adhesion to inorganic substrates and/or metal electrodes, making it substantial for optoelectronic devices and other engineering applications [15].

The major motivation of this study is to explore the polymer (P(VDC-co-AN)) primarily using the IGC techniques for surface thermodynamic properties. Besides, visual traits, such as intricate patterns, texture analysis, cross-sectional profiling, angular and radial spectra, particle area, directionality estimates were analyzed through the CVIP technique. Furthermore, in an economic stand-point, image analysis has its advantage, such as the solitary requirement of limited images with a decent resolution yet providing efficient analysis of visual traits with low cost and minimum human interference [16,17,18,19]. The usage of an expensive TEM imaging machinery would do the same by exhibiting the high definition imagery of the polymer. However, they lack in observing the intricate patterns and further visual traits, which, in most cases, are out shadowed by the imagery instrumental noise [20,21,22,23]. The real discussion is: how good is the exploration of this commercially useful polymer (P(VDC-co-AN)) through combined techniques from two relatively diverse fields of research CVIP & IGC? This question is partially elaborated through exhibiting the results and observations found by employing IGC and CVIP techniques on polymer (P(VDC-co-AN)) in its pure form. Eventually, the vicinity of the study is confined to characterize the surface thermodynamics of the polymer (P(VDC-co-AN)) using IGC techniques while also exhibiting a few of its visual traits when attempted to analyze using CVIP techniques. In the result-oriented perspective, this study aims to consolidate all of the aspects of IGC based polymer surface characterization as the main contribution. Nevertheless, this work also attempts to give some details of polymer’s visual traits from the image processing point of view in the interest of contributing towards future polymer studies in terms of employing image processing techniques on not so clear images, yet exploring the pure polymer (P(VDC-co-AN)) from a visual standpoint.

### 1.2. Inverse Gas Chromatography

Inverse Gas Chromatography is an effective technique for characterizing materials to investigate their physicochemical properties [24]. IGC technique is considered as a feasible approach due to its ability to determine surface properties of solids in crystalline and amorphous structures within various forms, such as powder, films, and fibers [25]. Besides having similarity in the principle to that of analytical gas chromatography techniques, IGC bears a unique phenomenon of placing the material in a column with a known probe vapor used to facilitate information on the surface of the material [26,27]. The range of properties such as entropy and enthalpy of adsorption, dispersive surface energy, solubility parameters, and many more aspects can be characterized while using the IGC technique [28]. The information obtained from the IGC experimentation enables researchers to exploit the true potential of the materials and unveil its usefulness in industrial applications and other fields of research. In this paper, the dispersive component and specific component of surface free energy have been evaluated using Schultz and Dorris–Gray methods [29,30,31]. The surface character value and the Lewis acid-base parameters were also evaluated by the Schultz method to investigate the nature of the surface of the polymer.

### 1.3. Computer Vision and Image Processing

The advancements in image analysis and accessibility of image data contributed vast growth in the field of technologies, such as Computer Vision and Image Processing (CVIP) [32,33,34]. Analogous to this, improvements in instrumentations related imaging techniques, such as Scanning Electron Microscopy (SEM) and Transmission Electron Microscopy (TEM) added an unprecedented precision to the fields of research, such as Inverse Gas Chromatography (IGC) and Nanotechnology. Exploiting such technological advancements in favor of scientific research has always been a conventional practice for the researchers [35]. As of two decades, computer vision and image processing have made their way into every possible division of research, including medicine, artificial intelligence, chemistry, physics, automobile, and agriculture [36,37,38,39,40]. The crucial aspect that motivated this interdisciplinary collective study is the ability to handle images and observe significant traits of the physical substance from various perspectives. As far as the polymer materials are concerned, features, such as intricate patterns of the substance in its pure form, surface texture, area measurements of the lumps, and ridges, are the traits that can be explored while using the image processing techniques. In this study, the SEM image analysis of (P(VDC-co-AN)) polymer has been carried out in order to determine the visual patterns, particle area distribution, particle directionality, and cross-sectional profiling of particle in both overview (500 μm) and portrait modes (200 μm and 50 μm). All of these visual elements were observed through a series of CVIP techniques, such as segmentation, thresholding, detection of feature maxima, texture analysis, etc.

**Figure 1:**
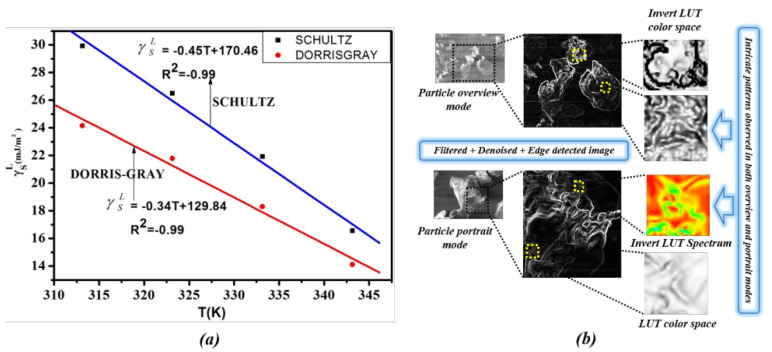
Inverse Gas Chromatography (IGC) surface thermodynamic characterization and Computer Vision and Image Processing (CVIP) Scanning Electron Microscopy (SEM) image analysis: (**a**) (P(VDC-co-AN)) surface free energy versus temperature using IGC methods. (**b**) Intricate visual patterns on the pure polymer surface observed using CVIP SEM image analysis.

A survey of the literature revealed that the surface thermodynamic characterization using IGC, as well as SEM image analysis on a pure form of (P(VDC-co-AN)) using CVIP techniques has not been reported earlier. On a whole, the paper attempts to explore the polymer (P(VDC-co-AN)) primarily using the IGC techniques for surface thermodynamic properties and secondarily employing customized CVIP pipelines for investigation of visual traits. The Figure 1 below illustrates the scenario of exploring the polymer (P(VDC-co-AN)) using IGC for surface thermodynamic characterization and CVIP methodologies for possible visual traits. The major highlights of the paper are as follows:

- Surface thermo-dynamic characterization of the polymer (P(VDC-co-AN)) has been carried. IGC attributes, such as London dispersive surface energy, Gibbs free energy, and Guttman Lewis acid-base parameters, were estimated.
- Visual traits, such as intricate patterns, surface morphology, texture/roughness, particle area distribution ($D_{A}$), directionality ($D_{P}$), mean average particle area ($\mu_{avg}$), and mean average particle standard deviation ($\sigma_{avg}$), were investigated using CVIP techniques on SEM images of the polymer in its purest form.

The rest of this paper is organized, as follows. Section 2 elaborates on the IGC experimental setup and SEM image acquisition used in this study. Section 3 describes the detailed calculation of IGC attributes, such as London dispersive surface energy, Gibbs free energy, and Guttman Lewis acid-base parameters. Section 4 illustrates the experimental analysis of polymer visual traits while using CVIP techniques. Section 5 presents the results and discussions corresponding to the IGC Surface thermo-dynamic characterization and CVIP based SEM image analysis. Finally, this paper is concluded in Section 6 with a summary.

## 2. Experimental Setup

### 2.1. IGC Experimental Setup

The IGC experimental analysis has been studied by using the AIMIL (model 5700, AIMIL Ltd., New Delhi, India) gas chromatography with dual column setup and fixed with FID detector. The IGC reading measurements were carried out at constant oven temperature at 10 ${}^{\circ }$C intervals and the range is set from 313.15–343.15 K. The P(VDC-co-AN) used in this study molecular weight, $\bar{M_{n}}=80,000$∼150,000 was purchased from Sigma–Aldrich Pvt. Ltd. (USA) and directly used for the preparation of the column packing. An exact amount of 0.8752 grams of polymer is packed directly into the column with inert component as helium gas. The probes n-alkanes (C5–C10), dichloromethane (DCM), Trichloro methane (TCM), acetone (AC), diethyl ether (DEE), ethyl acetate (EA), and tetrahydrofuran (THF) were analytical grade chemicals purchased from S.D Fine and Merck. The sieved P(VDC-co-AN) particles with 150–180 μm diameter were employed in column packing. The utilized stainless steel column (3 mm internal diameter and 30 cm length) was purchased from NUCON and the column was cleaned multiple times with acetone and methanol. The column is dried in the hot air oven. The sieved P(VDC-co-AN) was directly packed with the aid of mechanical vibrator and both ends of the column are filled with glass wool. Furthermore, the column was conditioned for 10 h under the continuous flow of nitrogen. The retention time ($t_{R}$) was measured using a Hamilton syringe 0.1 μL of each solute that was injected onto the P(VDC-co-AN) surface. The injection process for each solute was carried out three times on the P(VDC-co-AN) column. The average of all the three cycles was considered to calculate the net retention time.

### 2.2. SEM Image Acquisition

**Figure 2:**
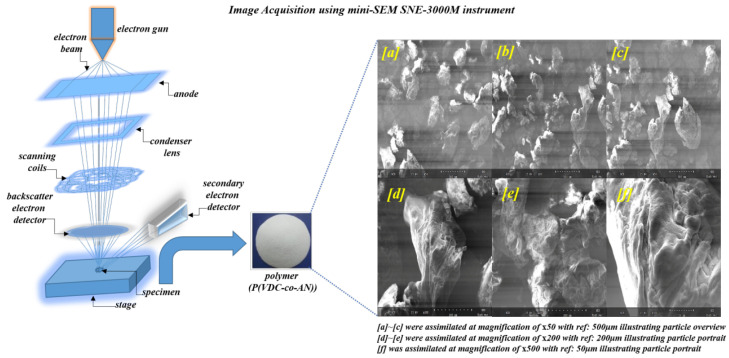
Image acquisition of polymer (P(VDC-co-AN)) using mini-SEM SNE-3000M instrument at different resolutions (overview mode: ×50, portrait mode: ×200 and ×500).

The SEM images were obtained using SNE-3000M mini-SEM instrument and they were analyzed using image processing software, such as OpenCV library [41], MATLAB [42], and Mathematica [43]. Languages, such as C++, C, and Wolfram, were employed to code the algorithms, such as thresholding, segmentation, and texture analysis on a Windows PC with NVIDIA GeForce GTX 980Ti Graphics Processor Unit (GPU). Properties, such as the distribution of area ($D_{A}$) and particle directionality ($D_{P}$) and cross-sectional profiling, were investigated using open-source image processing software, such as ImageJ [44] and Scanning Probe Image Processing (SPIP) [45]. Microsoft Excel and Origin7 [46] have been employed for pre-processing and post-calculations of polymer data. Various parameters involved in the texture analysis, directionality analysis, image enhancement, thresholding, and segmentation algorithms were fine-tuned to attain the best adaptive scaling on pixel-wise visual patterns that can reveal the finest patterns in the SEM image of the polymer (P(VDC-co-AN)). Certain arbitrary values have been chosen for better visualization to eliminate outliers and to enhance hidden patterns, under such circumstances a clear illustration has been documented in a pictorial representation regarding the effects imposed by those arbitrary values on the visual patterns. Figure 2 below illustrates the image acquisition of polymer (P(VDC-co-AN)) using mini-SEM SNE-3000M instrument at different resolutions (overview mode: ×50, portrait mode: ×200 and ×500).

## 3. IGC Surface Thermo-Dynamic Characterization

IGC technique has been used to determine the net retention volume, $V_{N}$ according to the following equation:

(1) $$V_{N}=\left(t_{R}-t_{0}\right)FJ\left(\frac{P_{o}-P_{W}}{P_{o}}\right),$$

where $t_{R}$ is the retention time of a probe, and to is the retention time of methane, *F* is the flow rate of carrier gas, $P_{W}$ is the saturated vapor pressure of water at ambient temperature, $P_{o}$ is the atmospheric pressure, and *J* is the James Martin correction factor.

The dispersive surface free energy, $\gamma_{S}^{d}$ of (P(VDC-co-AN)) has been calculated following Dorris–Gray equation [30]:

(2) $$\gamma_{S}^{d}=\frac{1}{4\gamma_{CH_{2}}}{\left[\frac{RT\ln\left(\frac{V_{N,n+1}}{V_{N,n}}\right)}{N\cdot a_{CH_{2}}}\right]}^{2},$$

where $\gamma_{CH_{2}}$ is the surface dispersive free energy of solid material containing only methylene groups (e.g., linear (P(VDC-co-AN))), and it is calculated at any temperature *t* (${}^{\circ }$C) with the Equation (3) stated in [47]:

(3) $$\gamma_{CH_{2}}=35.6-0.058t,$$

*N* is Avogadro’s number, *R* is gas constant, *T* is column temperature, and $a_{CH_{2}}=6{\overset{{}_{\circ }}{\;A}}^{2}$ is the cross-sectional area of adsorbed methylene group. $V_{N,n+1}$ and $V_{N,n}$ are the net retention volumes of n–alkanes with carbon number n+1 and *n*, respectively.

Alternatively, the dispersive surface free energy has been calculated following Equation (4) proposed by Schultz et al. [29].

(4) $$RT\ln V_{N}=2Na{\left(\gamma_{l}^{d}\right)}^{1/2}{\left(\gamma_{S}^{d}\right)}^{1/2}+K,$$

where *a* and $\gamma_{l}^{d}$ are the cross-sectional area and dispersive free energy of the adsorbate. The $\gamma_{S}^{d}$ of (P(VDC-co-AN)) can be evaluated using slope values obtained from a linear plot drawn between $RT\ln V_{N}$ versus $a{\left(\gamma_{l}^{d}\right)}^{1/2}$ of n-alkanes.

The specific component of surface free energy, $\Delta G_{a}^{S}$ values for the polar probes are evaluated while using Equation (5)

(5) $$-\Delta G_{a}^{S}=RT\ln\left(\frac{V_{N}}{V_{N(ref)}}\right).$$

The specific components of enthalpy of adsorption $\Delta H_{a}^{s}$, entropy of adsorption $\Delta S_{a}^{S}$, the Lewis acid-base parameters $K_{a}$, and $K_{b}$ are obtained using Equations (6)–(7)

(6) $$-\frac{\Delta G_{a}^{S}}{T}=\frac{-\Delta H_{a}^{S}}{T}+\Delta S_{a}^{S},$$

(7) $$-\frac{\Delta H_{a}^{s}}{AN^{*}}=K_{a}\left(\frac{DN}{AN^{*}}\right)+K_{b},$$

where $AN^{*}$ and DN are Guttmann’s modified acceptor and donor numbers, respectively. $AN^{*}$ and DN values along with the physical property data for the probes are given in Appendix A, Table A1. The *a* and $\gamma_{l}^{d}$ values for different probes were retrieved from previous IGC studies [47].

## 4. Image Analysis of (P(VDC-co-AN)) Visual Traits Using CVIP Techniques

**Figure 3:**
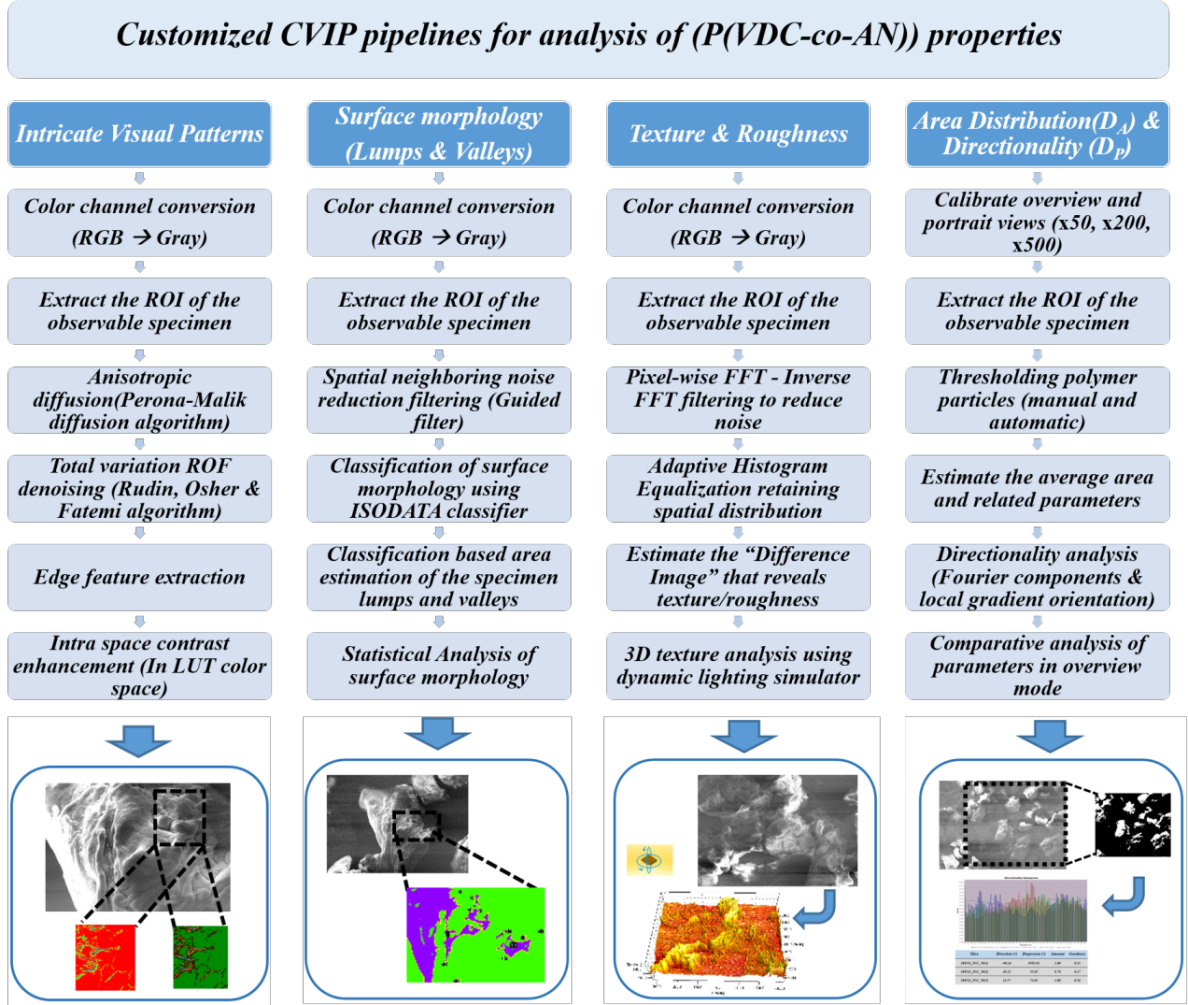
Customized CVIP pipelines for image analysis of (P(VDC-co-AN)) polymer for visual traits.

In the customized pipeline of image analysis, the images obtained from any imaging instrument bear a certain amount of uncertainty in pixel accuracy due to instrument-induced noise [48]. To reduce such noise outliers, image enhancement techniques, such as filtering, denoising, feature enhancement, etc., were employed. These techniques act as a primary image purifier for later stages of image analysis techniques, such as contour detection, intricate pattern analysis, classification of structural lumps, and surface area distribution. Filtering techniques of three types, such as anisotropic diffusion [49] with total variation denoising [50], edge preserving guided filtering [51] with spatial neighboring noise removal, and Fast Fourier Transform (FFT) filtering [52] with Adaptive Histogram Equalization (ADE) were employed in a customized pipeline model for image enhancement and noise removal. The images were analyzed based on the various customized pipelines and each pipeline has its unique pros and cons in correspondence to various specimen properties. Example: use of customized anisotropic diffusion with variation denoising and outliers removal pipeline produces higher peak signal to noise ratio (PSNR) and good structural similarity index measure (SSIM) as compared to the other techniques, which imply the ability to view the specimen in less noise environment. In contrast, the same image might have a relatively minor consideration in the context of anomaly detection using directional transforms. Given these aspects, suitable approaches have been chosen, depending upon requirements and scenarios of certain analyses. The Figure 3 below illustrates the customized CVIP pipelines for SEM image analysis of (P(VDC-co-AN)) polymer for visual traits.

### 4.1. Intricate Visual Patterns

The visual traits can be characterized into various categories, such as surface roughness, structural visual patterns, texture forms, directionality, cross-sectional profiling and area distribution, etc. The intricate visual patterns of the polymer can be observed through the application of edge contouring on an anisotropic diffusion that nis filtered with a noise-reduced image. The pipeline comprises of denoising, filtering, and edge contour as pre-processing and intra color adjustments as post-processing. The anisotropic diffusion filtering proposed by Perona–Malik [53] was employed as a part of the pre-processing. Furthermore, post-processing techniques, such as intra color space contrast adjustments, were employed using the ImageJ toolbox for a better perception of the visual patterns on the surface of the polymer. The major pre-processing was carried out using the anisotropic diffusion filtering with a noise reduction step, as depicted in the Equation (8), below:

(8) $$\frac{\partial I}{\partial t}=\mathrm{div}\left(c\left(x,y,t\right)\nabla I\right)=\nabla c\cdot \nabla I+c\left(x,y,t\right)\Delta I,$$

where Δ,∇ denotes Laplacian and gradient, respectively. div() denotes the divergence operator and c(x,y,t) represents the diffusion coefficient proposed by Perona–Malik, which controls the magnitude of image gradient, preserving the edges and details.

### 4.2. Surface Morphology-Lumps and Valleys

The surface morphology of the polymer includes several structural elements in the image domain, such as shape, surface voxel distribution, etc. These traits were observed through a series of pre-processing and post-processing techniques, such as spatial neighboring noise reduction using guided filtering: a pioneering technique of edge preservation filtering. The filtering technique that was proposed by K. He et al. [54] was employed to obtain the initial edge preserved imaging for later pipeline processing. The pre-processed image has been classified using an ISODATA classifier [55] based on classes observed on the surface of the specimen namely, lumps, valleys, and high-intensity flat surfaces. The underlying properties of any distributed model can be analyzed while using the ISODATA classifier due to its unsupervised clustering capabilities. The polymer specimen surface has been classified using the ISODATA classifier technique in ImageJ automated software and then the lump areas were analyzed and marked statistically. The major pre-processing was carried out using the edge-preserving guided filtering, as shown in the Equation (9), below:

(9) $$q_{i}=\frac{1}{\left|\omega \right|}\sum_{k:i\in \omega_{k}}\left(a_{k}I_{i}+b_{k}\right)={\bar{a}}_{i}I_{i}+{\bar{b}}_{i},$$

where $q_{i}$ is filtered output and *I* denotes guidance image. $\left(a_{k},b_{k}\right)$ are some linear coefficients and $\omega_{k}$ is window centered at *k*.

### 4.3. Texture and Roughness

The frequency-domain representation of a specimen exhibits the spatially distributed properties, such as texture and roughness. Image processing techniques such as Fast Fourier Transform (FFT) were employed to ensure the presence of frequency representation of the polymer specimen by purging the high-frequency intensities (noise outliers). Moreover, the image gets reverted from the frequency domain to the image domain using an inverse FFT technique and equalized using Adaptive Histogram Equalization (AHE). Additionally, a non-trivial yet novel concept of the difference image ($I_{diff}$) has been formulated while using Equation (10) to customize the analysis step even more substantial to observable texture and roughness features.

(10) $$I_{diff}=P_{3D}\left[I_{xy}-FFT_{AHE}\left(I_{xy}\right)\right],$$

where $I_{diff}$ represents the difference image, $P_{3D}$ is three-dimensional (3D) projection operator, $I_{xy}$ denotes the original image and $FFT_{AHE}\left(I_{xy}\right)$ denotes frequency domain filtered and histogram equalized image.

### 4.4. Area Distribution (DA) and Particle Directionality (DP)

In the estimation of structural aspects, such as area distribution and particle directionality, SEM images were analyzed with open-source software, such as SPIP and ImageJ. The polymer SEM particle overview image has been considered for the estimation of area distribution and particle directionality [56]. The selected Region of Interest (ROI) was processed accordingly for the estimation of the thresholding as a stage-1 in the pipeline. The threshold classified image is then manually refined by color space edge enhancement for robust segmentation revealing the fine contours as a stage-2. The particle directionality analysis (DP) of the particle overview has been made while using the ImageJ toolkit in two different scenarios, such as directionality based on Fourier components and directionality based on local gradients orientation. Furthermore, the average distribution of particle area has been estimated and statistics were applied to retrieve the spatial distribution, such as average mean particle area, the standard deviation in terms of $\mu m^{2}$ were estimated using Equations (11)–(12):

(11) $$\left(\mu_{avg}\right)=\frac{\sum_{k=1}^{N}\mu_{k}}{N},$$

(12) $$\left(\sigma_{avg}\right)=\sqrt{\frac{1}{N}\sum_{k=1}^{N}{\left(x_{k}-\mu_{k}\right)}^{2}},$$

where $\mu_{avg}$ and $\sigma_{avg}$ are average mean and average standard deviation of the particle area distribution with *N* particles (N>k), *k* being the particular index of the particle *x* of individual mean $\mu_{k}$.

## 5. Results and Discussions

### 5.1. IGC Study on Polymer Surface Characterization

**Figure 4:**
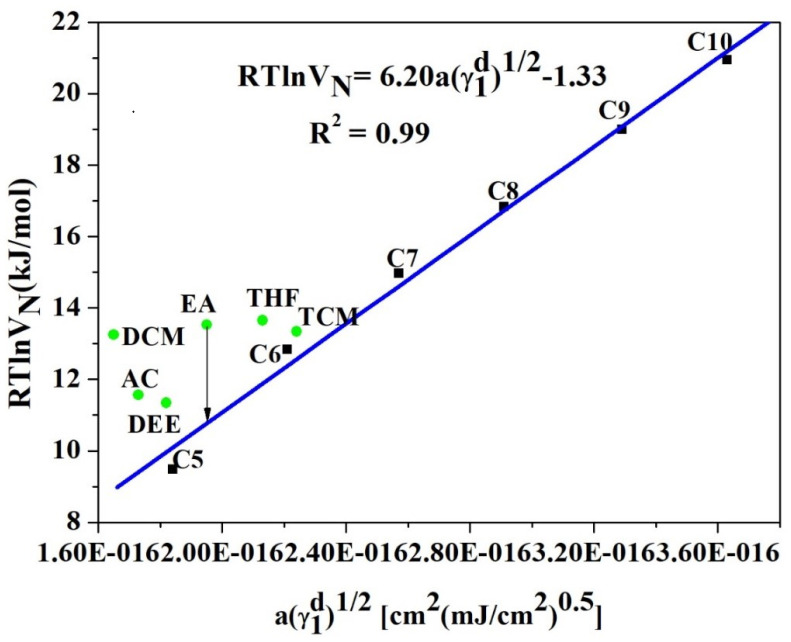
$RT\ln V_{N}$ versus $a\sqrt{\gamma_{l}^{d}}$ for n-alkanes and polar probes for (P(VDC-co-AN)) surface at 313.15 K. (Schultz).

The $RT\ln V_{N}$ values of n- alkanes and polar probes were measured for the (P(VDC-co-AN)) at temperatures ranging from 313.15 K to 343.15 K. The $RT\ln V_{N}$ values are linearly decreased with the increase in temperature. The $RT\ln V_{N}$ values are shown in Table 1 and the variation of $RT\ln V_{N}$ versus $a\sqrt{\gamma_{l}^{d}}$ plot is illustrated in Figure 4, below.

**Figure 5:**
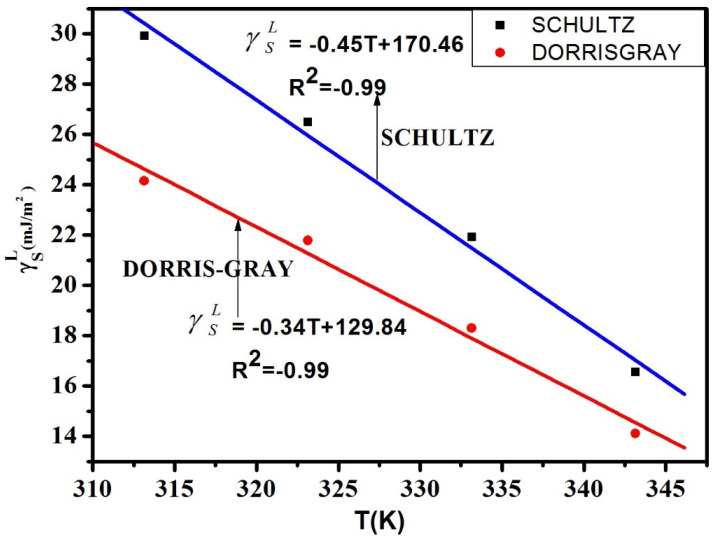
Comparison Plots of dispersive surface free energy versus temperature for (P(VDC-co-AN)) surface.

The London dispersive surface free energy $\gamma_{S}^{L}$ was calculated by Schultz and Dorris-Gray methods using Equations (2)–(4). The $\gamma_{S}^{L}$ values are decreasing linearly with the increase of temperature in both methods, and the correlation coefficient is reliable. The maximum $\gamma_{S}^{L}$ is $29.93\;\mathrm{mJ}/m^{2}$ in Schultz and $24.15\;\mathrm{mJ}/m^{2}$ in Dorris-Gray method. The $\gamma_{S}^{L}$ values are shown Table 2 and the variation of temperature versus $\gamma_{S}^{L}$ values are shown in Figure 5 below. The $\gamma_{S}^{L}$ value in between the temperature range of (313.15–343.15 K) signifies the surface structural changes of the solid material and its allowance to the penetration of the probe molecules. As observed, the temperature gradient of $\gamma_{S}^{L}$ is negative, which indicates the rise in the distance of accession between the (P(VDC-co-AN)) molecules with the escalation in the temperature. According to the London expression, the dispersive energy is inversely proportional to the sixth power of the distance of separation between the molecules. Therefore, with an escalation in the temperature, the dispersive energy increases, and the distance of dissolution between molecules decreases.

**Figure 6:**
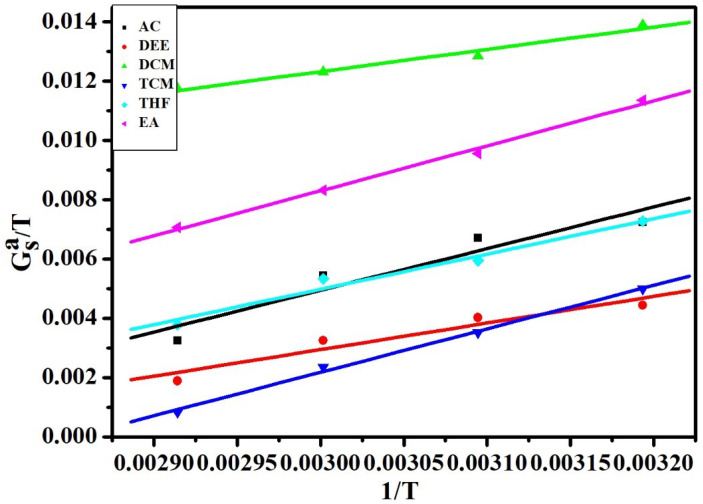
The plot of $-\Delta G_{a}^{S}/T$ versus 1/T on (P(VDC-co-AN)) (Schultz).

The results obtained from the Schultz method and Dorris–Gray method clearly illustrate the increase and gradual decrease of the London dispersive surface energy value with an increase in temperature. This discrepancy may be ascribed to the values of the dispersive surface tensions of the liquid probes $\gamma_{S}^{L}$. However, as compared to the Dorris–Gray method, the Schultz method is the most substantial for the estimation of London dispersive surface energy, $\gamma_{S}^{L}$ at elevated temperatures [47]. Nevertheless, the Dorris-Gray method is applicable at ambient temperatures with a known temperature dependency on $\gamma_{S}^{L}$. Commonly, the strength of acid-base communication between the adsorbent and polar probe determines the numerical values of $\Delta G_{a}^{S}$. Furthermore, the regular area of the probe’s adsorption on the drug material has some influence on the $\Delta G_{a}^{S}$ values. The elevation in temperature effects Gibb’s surface energy of adsorption to be further negative. The Gibb’s surface free energy $\Delta G_{a}^{S}$ values for the polar probes were obtained by the Schultz’s method while using Equations (5)–(7) respectively. The $\Delta G_{a}^{S}$ versus temperature variation is shown in Figure 6.

The $\Delta G_{a}^{S}$ values indicate that the polar probes interacted strongly with the polymer surface. In (P(VDC-co-AN)), the lowest $\Delta G_{a}^{S}$ value observed in the DCM solvent and the highest $-\Delta G_{a}^{S}$ value was observed in EA. The order of increase is shown below.

DCM<DEE<THF<AC<TCM<EA

**Figure 7:**
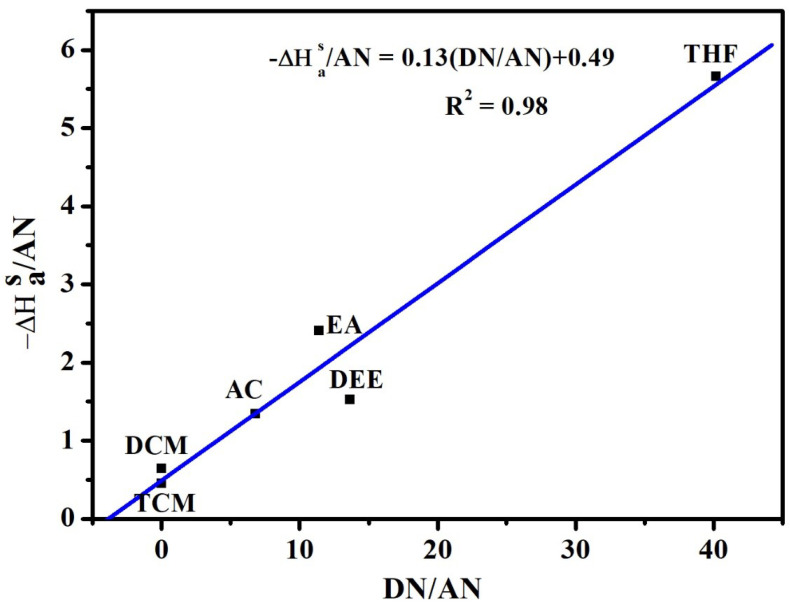
The plot of $\Delta H_{a}^{S}/\mathrm{AN}$ versus DN/AN for the surface of (P(VDC-co-AN)) (Schultz).

Based on Equation (6), the polymer (P(VDC-co-AN)) variation of $-\Delta G_{a}^{S}/T$ versus 1/T is found to be linear and, hence, a statistical fit has been applied to evaluate $\Delta H_{a}^{s}$ and $\Delta S_{a}^{S}$. The $\Delta H_{a}^{s}$, $\Delta S_{a}^{S}$ values, and the correlation coefficient $R^{2}$ are given in Table 3. Furthermore, $\Delta H_{a}^{s}$ values along with Guttmann Lewis acid-base parameters have been used in Equation (7) to evaluate their Lewis acid-base parameters. The variation of $\Delta H_{a}^{S}/\mathrm{AN}$ and DN/AN has been shown in Figure 7.

The $K_{a}$ and $K_{b}$ values are obtained from the slope and intercept of the linear plot, respectively, and are shown in Table 4. The correlation coefficient ($R^{2}$) obtained from the calculation appears to be significant. The $K_{a}$ and $K_{b}$ values are related to the amount of acidic and basic sites on the surface of the polymer (P(VDC-co-AN)). The $K_{a}$ value is 0.13, $K_{b}$ value is 0.49 and the correlation coefficient $R^{2}$ is 0.98.

The surface character ‘*S*’ represents the overall character of the polymer surface. The surface *S* value is 3.77, which is higher than 1, that implies the overall surface character is considered to be basic, if less than 1, the surface is considered as acidic. Therefore the overall character of the polymer (P(VDC-co-AN)) is considered basic. Accordingly, the polymer (P(VDC-co-AN)) can interact with strongly acidic media. The $K_{a}$ and $K_{b}$ values were associated with the quality and nature (acidic or basic) of the sites of (P(VDC-co-AN)) surface. The results revealed that the (P(VDC-co-AN)) polymer surface contains more basic sites than acidic sites and, hence, can closely associate in acidic media.

### 5.2. CVIP Study on Polymer Visual Traits

**Figure 8:**
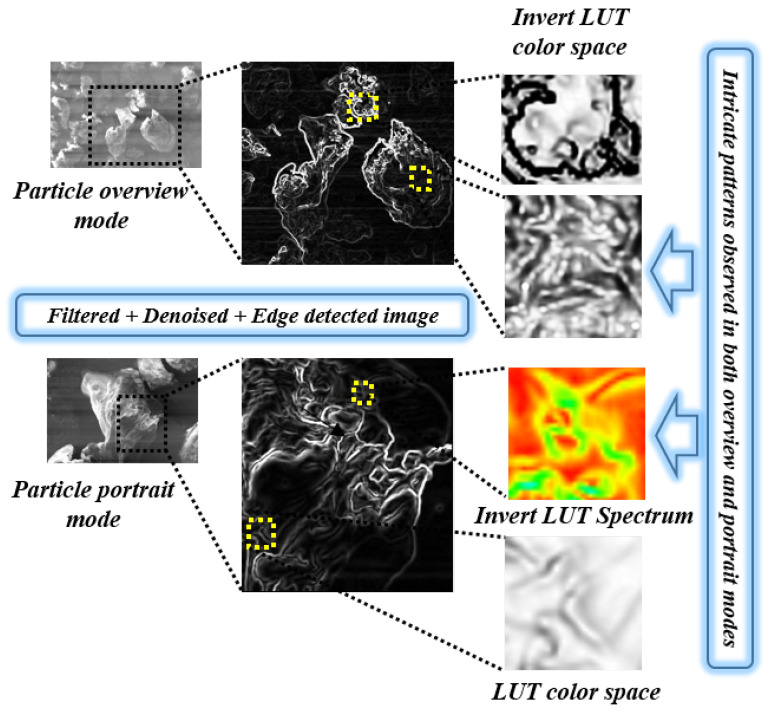
Customized image analysis pipeline for exploring visual intricate patterns in (P(VDC-co-AN)) polymer.

The non-linear space-variant transformation of the polymer specimen was examined by using anisotropic diffusion filter. The retrieved image attributes were denoised and edge detected to observe the polymer’s intricate patterns shown in Figure 8. The illustrated patterns were unable to observe in the presence of the instrumental noise and imaging anomalies. However, when customized pre-processing is applied, the patterns unveil the real surface perceptions of the polymer in both overview and portrait views. The patterns observed in Figure 8 illustrate the elaborate details of the polymer, such as quills and spikes, which otherwise cannot be seen using low-level imaging instruments. Furthermore, the visual intricate patterns on the pure form of polymer surface enable the chemical enthusiasts to obtain intuition regarding the binding possibilities in fusion with new materials.

**Figure 9:**
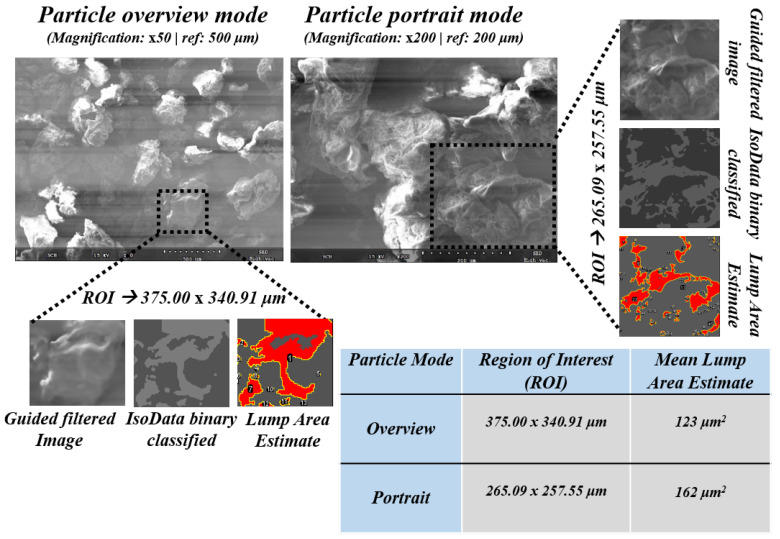
Customized image analysis pipeline for estimation of polymer surface morphology attributes (mean lump area).

**Figure A1:**
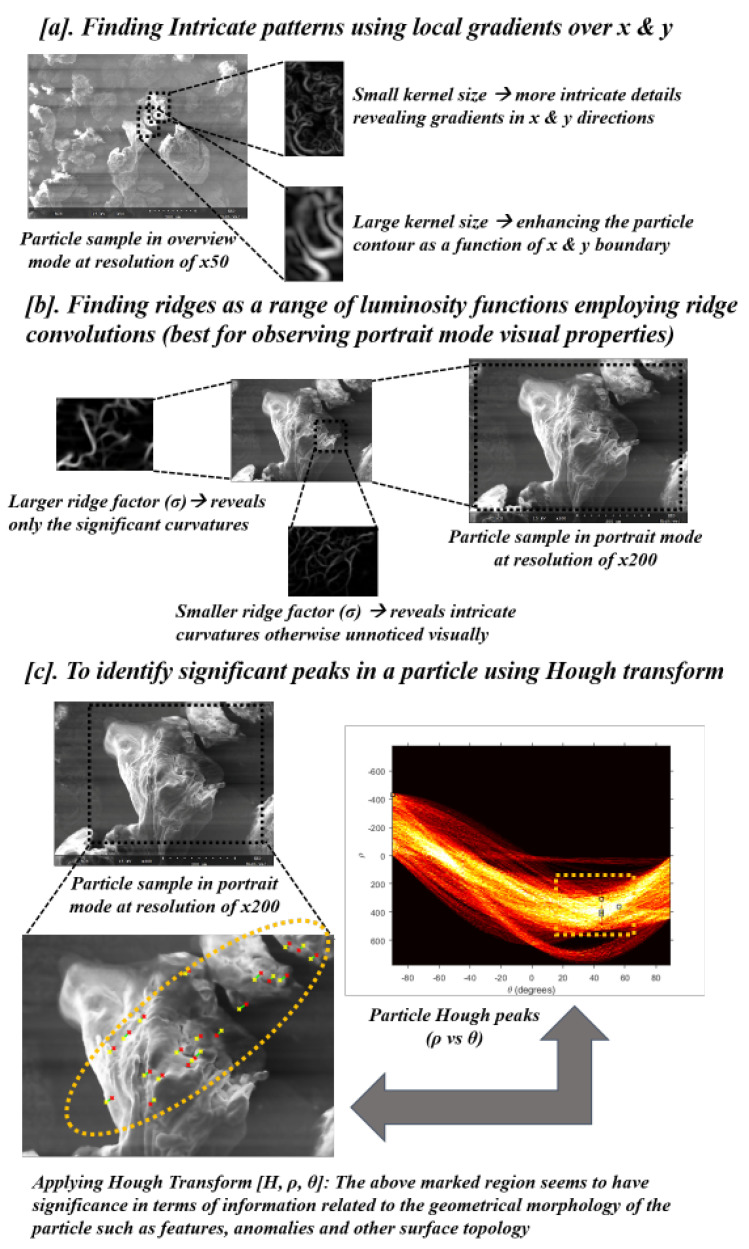
Detecting intricate patterns and substantial peaks: (**a**) Investigation of polymer visual intricate patterns using gradient filter. (**b**) Observation of polymer visual intricate patterns using ridge filter. (**c**) Identification of significant peaks in the polymer image specimen using Hough transform.

The ISODATA classification has been employed on both overview particle image and portrait image. The SEM was calibrated to set the dimensions as per their captured resolutions and reference scales, such as 500 μm, 200 μm, 50 μm. Moreover, chosen ROI dimensions of 375.00 × 340.91 μm and 265.09 × 257.55 μm were applied on the particle overview and particle portrait, respectively. Essential morphological features, such as edges, were preserved by utilizing filtering techniques, such as guided filters, and further reducing the neighborhood pixel-level noise. The surface of the specimen has been classified into lumps and valleys along with the estimation of their distribution attributes such as surface voxels, area, etc. The clear idea of distribution in the population of lumps and valleys can affect the surface chemical properties of the associating chemical in the context of composite research. As a statistical estimate, the mean lump area estimates were calculated for both overview and portrait modes as 123 μm${}^{2}$ and 162 μm${}^{2}$, respectively, which are depicted in Figure 9. Additionally, visual traits of the polymer were extracted using extended methodologies, such as gradient filters and ridge convolutions, which are used to reveal the elaborate lumps and contour details of the specimen. Additionally, the significant peaks in the polymer specimen can help the chemists to estimate the best possible use of the polymer for future composite reactions. The Hough transform has been employed to identify the significant peaks in the specimen and corresponding results were illustrated in Figure A1 in the Appendix B.

**Figure 10:**
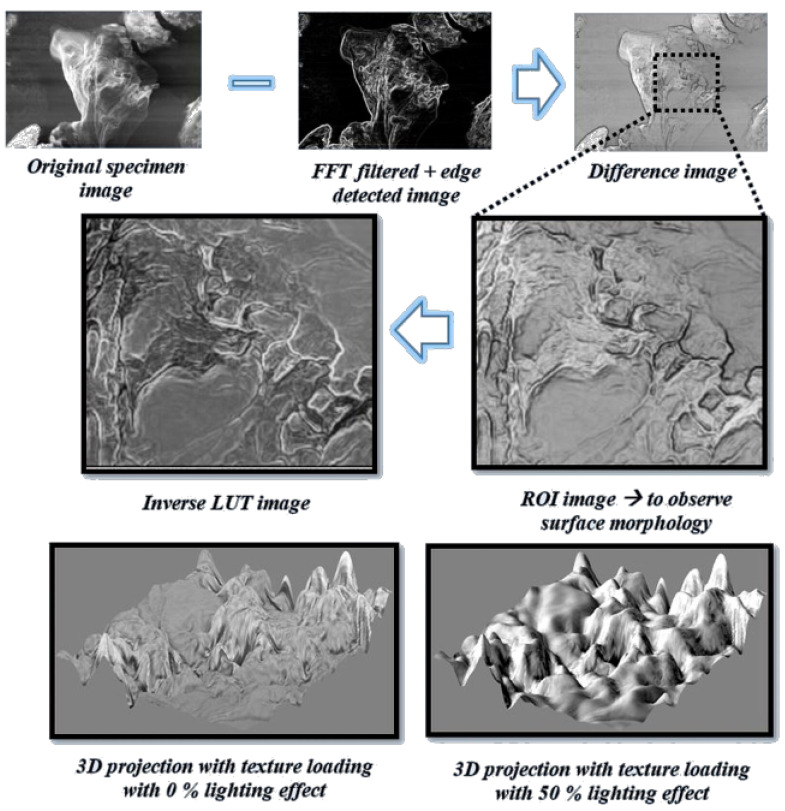
Customized difference image pipeline for investigating texture and three-dimensional (3D) roughness with lighting variations.

Although the resultant visuals obtained from the inspection of the polymer in the image domain were prominent, it is essential to also examine the visual footprint of the polymer (P(VDC-co-AN)) in the frequency domain. In that context, the SEM images were processed while using a customized pipeline of consequent frequency-domain noise removal techniques. The images were filtered using the fast Fourier transform filter which yields the frequency domain component of the image, which comprises all of the frequency-wise distribution analogous to the pixel-wise in the image domain. This is followed by an inverse fast Fourier transform recovering back the image obtained from the frequency components which denoised the image in the frequency domain by retaining the most crucial part of the image. Additionally, to compensate for any spatial irregularities that are caused by the transition of image-to-frequency domain and back- the spatial arrangement of intensity values were balanced using adaptive histogram equalization. Also, the concept of difference image has been formulated from the pixel-wise subtraction of FFT filtered histogram equalized image from the original specimen. This pipeline modeling was illustrated in Figure 10 with additional techniques, such as post-color transformation to Inverse LUT domain and the application of customized lighting simulator. The results obtained using these frequency-based image processing with lighting simulator aids the clear observance of the polymer surface morphology in 3D format. The adaptive histogram equalization method enhanced the image pixel intensities and high-frequency noise components are removed for better visualization. This can be observed in Figure 10 within the visualizations of 3D projection with texture loading. These results might aid the researchers in visually exploring the pure form of the polymer’s surface without employing expensive imaging machinery such as TEM.

**Figure A2:**
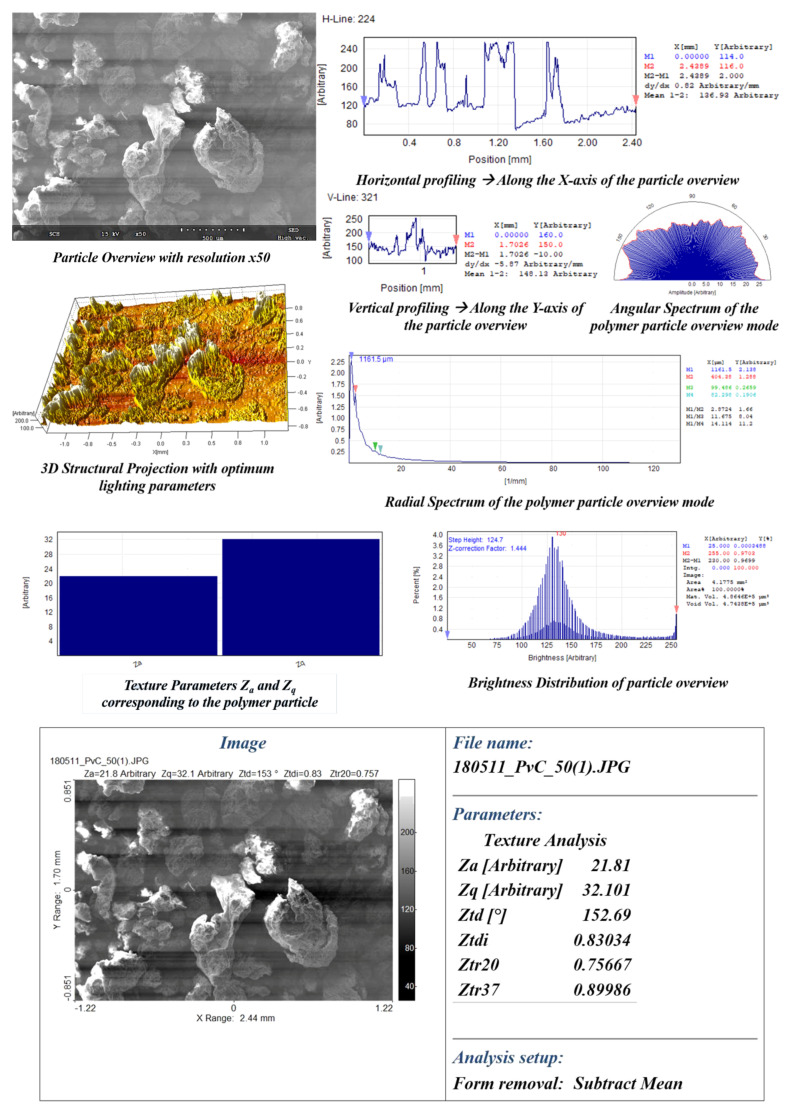
Investigation of texture properties, cross-sectional profiling, angular spectrum and 3D structural projection of the particle (in overview mode with resolution: ×50) using SPIP.

**Figure A3:**
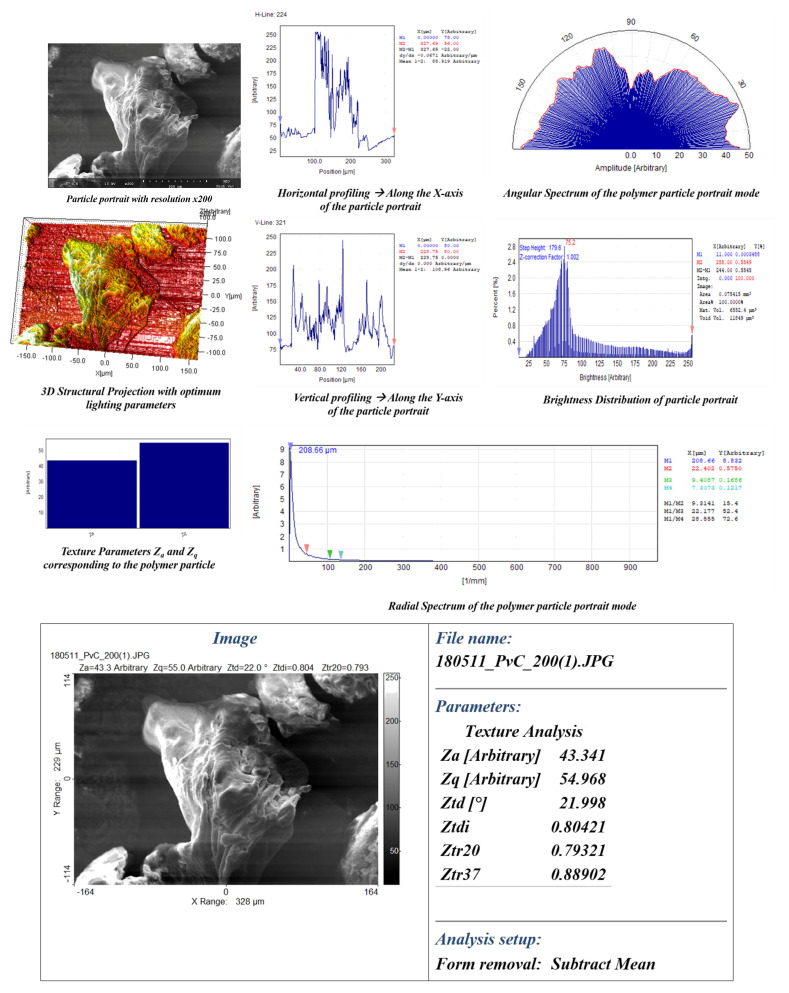
Investigation of texture properties, cross-sectional profiling, angular spectrum and 3D structural projection of the particle (in portrait mode with resolution: ×200) using SPIP.

**Figure A4:**
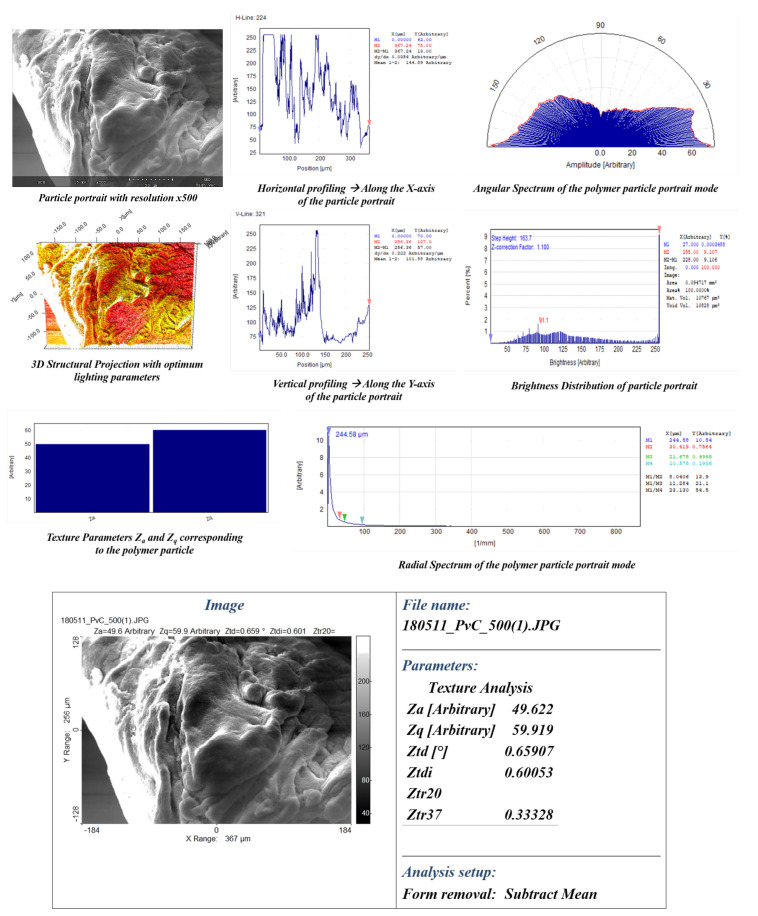
Investigation of texture properties, cross-sectional profiling, angular spectrum and 3D structural projection of the particle (in portrait mode with resolution: ×500) using SPIP.

Furthermore, properties such as texture parameters were estimated using the SPIP texture analysis toolkit; the results are illustrated in Figure A2, Figure A3 and Figure A4 in the Appendix B. Visual observations such as dual axis polymer surface profiling, angular and radial spectrum, texture analysis were estimated from particle overview and portrait modes. These results are shown in Figure A2, Figure A3 and Figure A4 assist the researchers to speculate on the texture-based traits of the pure polymer (P(VDC-co-AN)). In terms of statistical analysis, investigation on particle overview mode has been carried out to estimate the particle distribution in terms of area distribution ($D_{A}$) and particle directionality ($D_{P}$). The refined thresholding pipeline yielded a detection of (N=7) particles within a particle overview ROI of 381.68 × 442.75 μm. The average mean area of the ROI considered was estimated to be 157 μm${}^{2}$ and the average mean standard deviation was estimated to be 37.2 μm${}^{2}$.

**Figure 11:**
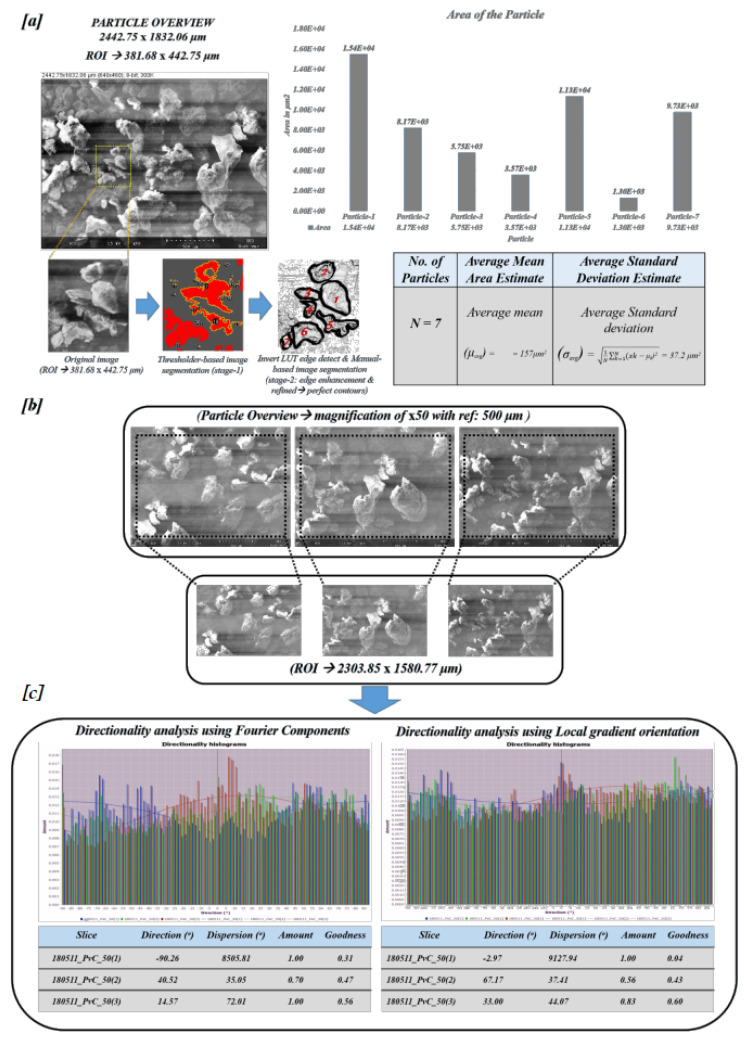
Particle area distribution and directionality analysis: (**a**) Statistical attributes ($\mu_{avg},\sigma_{avg}$) of particle area distribution. (**b**) Observation of polymer visual intricate patterns using ridge filter. (**c**) Directionality analysis using Fourier components and local gradient orientation.

Figure 11a depicts the clear analysis of the area distribution in the considered specimen along with the particle individual area estimates. The specimen’s overview modes in (max = 3) view with resolution ×50 has been pre-processed in order to extract the ROI of 2303.85 × 1580.77 μm and directionality analysis has been made using Fourier components and local gradient orientation. Figure 11b illustrates the directionality of the particles depicted in a plot with histogram bins with Direction (θ) in X-axis representing the center of the Gaussian fit and Amount in Y-axis represents the amount of directionality, which is calculated by the statistical parameter, such as standard deviation limit threshold. In the case of Fourier components, the peaks ranged between $-90^{\circ }$ to $+90^{\circ }$ among which major peaks can be observed between $+5^{\circ }$ to $+10^{\circ }$, which represents the polymer particle orientation at that bin in analogous to the overview specimen picture. In the case of local gradient orientation, the peaks can be observed between $0^{\circ }$ to $+5^{\circ }$ depicting the strong orientations in the particles. For adhesive applications and etching processes, the polymer surface directionality information could be of assistance towards employing polymer (P(VDC-co-AN)) in conjunction with other materials.

The visual patterns of the polymer can also be observed using some standard filters such as gradient filters that enhance the features of the image specimen in certain desirable directions (along x-axis & y-axis). Unlike the gradient filters, the ridge filters can employ the convolutions to work on the range of luminosity in a given image. Both the gradient filter and ridge filters have their fine-tunable kernel size and ridge factor (σ) respectively. With the smaller kernel size in the gradient filter, the intricate patterns can be observed in the specimen and with the larger kernel size, contours can be observed clearly. Likewise, the ridge filter outputs the significant curvatures in the image specimen when applied with a larger ridge factor and all of the intricate patterns can be observed with the smaller ridge factor. This phenomenon can be observed in the Figure A1a,b. The image specimen contains various pixel-wise features and one way to identify the significant features in the image domain is through the application of transforms, such as Hough transform. The Hough parameters (H,ρ,θ) are tuned such that the marked region in Figure A1c of particle portrait region appears to be significant due to the presence of Hough peaks. The additional image processing techniques were employed by the usage of examples provided by MATLAB and Mathematica repositories.

The visual traits of the polymer related to texture and surface roughness were investigated using a SPIP toolbox. The surface morphology of the polymer along with its cross-sectional profiling can be better explored using this toolbox. Additionally, special features in an image, such as angular spectrum and radial spectrum, were plotted along with the brightness distribution of the image specimen. Finally, texture parameters, such as Texture Average ($Z_{a}$) and Root Mean Square ($Z_{q}$) were plotted on an arbitrary scale and 3D structural projections with optimum lighting conditions were plotted for better understanding of the surface morphology. The above-stated traits were calculated and plotted for overview (×50) and portrait (×200, ×500) modes. The values of $Z_{td}{[}^{\circ }],Z_{tdi},Z_{tr20}$ and $Z_{tr37}$ represents texture direction in degrees, texture direction index, aspect ratio at 20% and 37% of the highest peak respectively. The above readings are illustrated in Figure A2, Figure A3 and Figure A4 for specimen resolutions of ×50, ×200 and ×500, respectively. These values represent the texture and roughness aspects of the polymer in various modes and resolutions and the angular spectrum represents the angular specification of high occurring peaks of texture. For better visualization, the 3D structural projection of the polymer can be observed in the respective depictions. The use of customized CVIP techniques aims to target and provide the insights of the polymer (P(VDC-co-AN)) surface observations, such as intricate patterns, surface morphology, area distribution, dimensionality, texture, and roughness analysis, which can be used to estimate the proper usage of the pure form of polymer (P(VDC-co-AN)) for future application and research purposes.

## 6. Conclusions

In this work, polymer (P(VDC-co-AN)) was systematically investigated using IGC and CVIP techniques. Using IGC attributes, such as London dispersive surface energy, Gibbs free energy, and Guttman Lewis acid-base parameters, were analyzed for the polymer. The London dispersive surface energy was calculated while using the Schultz and Dorris–Gray method. The values are decreased linearly with the increase of temperature. The maximum surface value of (P (VDC-co-AN )) is found to be 29.93 and 24.15 in both methods, respectively. The Guttman–Lewis acid-base parameter $K_{a},K_{b}$ values were estimated to be 0.13 and 0.49. The surface character *S* value is 3.77 and the correlation coefficient is 0.98, respectively. After the thermo-dynamic surface characterization, the (P(VDC-co-AN)) polymer overall surface character is found to be basic. The substantial results revealed that the (P(VDC-co-AN)) polymer surface contains more basic sites than acidic sites and, hence, can closely associate in acidic media. Additionally, the intricate visual patterns, texture traits, and particle distribution statistics were investigated through series of customized image processing pipelines on the pure polymer form an overview (×50) and portrait (×200, ×500) modes. This combined study of exploring the (P(VDC-co-AN)) polymer facilitated the better representation of its properties and traits both in chemical and visual perspective.

## Figures and Tables

**Table 1:** $RT\ln V_{N}$ values of n- alkanes and polar probes measured for the (P(VDC-co-AN)) at temperatures ranging from 313.15 K to 343.15 K.

Solutes	313.15 K	323.15 K	333.15 K	343.15 K
n-Pentane	7.40	7.61	7.41	–
n-Hexane	9.51	10.02	9.37	9.27
n-Heptane	10.92	11.50	12.22	10.42
n-Octane	12.48	13.10	13.84	11.82
n-Nonane	16.03	15.16	15.51	13.22
n-Decane	18.11	17.21	16.94	14.91
Acetone	17.88	18.48	18.61	16.28
Di-ethyl ether	17.70	17.87	17.84	16.02
Dichloromethane	17.26	17.51	17.56	15.61
Trichloromethane	19.82	19.62	19.27	16.66
Tetrahydrofuran	19.11	19.05	18.74	17.04
Ethyl acetate	19.32	18.45	18.37	16.98

**Table 2:** Dispersive surface free energy, $\gamma_{S}^{L}$ (${\mathrm{mJm}}^{-2}$) of (P(VDC-co-AN)).

T(K)	$\gamma_{S}^{L}$ (Schultz)	$\gamma_{S}^{L}$ (Dorris-Gray)
313.15	29.93	24.15
323.15	26.49	21.78
333.15	21.92	18.30
343.15	16.84	14.11

**Table 3:** The specific components of enthalpy of adsorption, $\Delta H_{a}^{S}$ and entropy of adsorption, $\Delta S_{a}^{s}$ for polar probes on (P(VDC-co-AN)).

Solutes	$-\Delta H_{a}^{S}$ (kJ mol${}^{-1}$)	$-\Delta S_{a}^{s}$ (kJ mol${}^{-1}K^{-1}$)	r
Acetone	14.07	−0.04	0.95
Di-ethyl ether	9.00	−0.02	0.96
Dichloromethane	7.49	−0.01	0.99
Trichloromethane	14.67	−0.04	0.99
Tetrahydrofuran	11.89	−0.03	0.99
Ethyl acetate	15.19	−0.03	0.99

**Table 4:** Lewis acid-base parameters, surface character and correlation coefficient of the polymer (P(VDC-co-AN)).

Method	$K_{a}$	$K_{b}$	*S*	$R^{2}$
Schultz et al.	0.13	0.49	3.77	0.98

## Appendix A. IGC Appendix

The values of $AN^{*}$ and DN along with the physical property data for the probes used in the IGC experiment are given in Table A1.

**Table A1:** Physical constants for probes used in IGC experiments.

Solute	$a{\left(\gamma_{l}^{d}\right)}^{0.5}\times 10^{-16}$ ${\mathrm{cm}}^{2}{\left(mJ/{\mathrm{cm}}^{2}\right)}^{0.5}$	*a* $\left({\mathrm{nm}}^{2}\right)$	AN (kJ/mol)	DN (kJ/mol)
n-Hexane	2.21	0.515	-	-
n-Heptane	2.57	0.570	-	-
n-Octane	2.91	0.630	-	-
n-Nonane	3.29	0.690	-	-
n-Decane	3.63	0.750	-	-
Acetone	1.73	0.425	10.5	71.4
Diethyl ether	1.82	0.470	5.88	80.6
Trichloromethane	2.24	0.440	22.7	0
Tetrahydrofuran	2.13	0.450	2.1	84.4
Ethyl acetate	1.95	0.480	6.3	71.8

## Appendix B. CVIP Appendix

During the process of image analysis, an extra set of customized image processing pipelines were used from existing algorithms such as ridge filtering, Hough transforms to observe possible outcomes retrieved from a noisy SEM image. The impact of kernel size and gradients in the context of enhancement filtering is shown in Figure A1a. Similarly, the impact of the ridge factor in conjunction with luminosity on the surface curvatures in the polymer image can be observed in Figure A1b. Additionally, the usage of Hough transforms on the portrait mode identified the particle peaks in the surface morphology of the polymer in the observed context. The resulting peaks in the form of polar coordinates are depicted in Figure A1c. In general, techniques such as these can be used to unveil the structural analogies which are often overshadowed by the instrumental noise. The SPIP toolbox has been employed to investigate the polymer texture and surface roughness in conjunction with the polymer surface morphology concerning the cross-sectional profiling (horizontal and vertical). The brightness distributions of the polymer image are analyzed using spectrum in angular and radial modes. These resultant plots are depicted in Figure A2 which are useful for understanding the curvatures of the surface in overview mode. A similar set of attributes were estimated for the portrait modes which were shown in Figure A3 and Figure A4 to have a side-by-side comparison of the cross-sectional profiling and spectra. The SPIP toolbox served as a great tool in calculating various numerical estimates for the texture and roughness of the polymer surface in overview and portrait modes. The $Z_{a}$ and $Z_{q}$ values estimated using the toolbox concerning portrait mode with resolution ×500 tend to have higher values indicating the roughness texture of the polymer surface. These supplementary visual observations retrieved from the SPIP image analysis toolbox appeared to offer additional information associated with that of the mainstream visual traits conversed in the main text and thus these supplementary results are organized into this CVIP related appendix section for further reference.
